# Robotic-assisted right colectomy with the DEXTER^®^ Robotic Surgery System: first prospective multicenter study

**DOI:** 10.1007/s10151-026-03385-7

**Published:** 2026-07-05

**Authors:** J. Pochhammer, T. Becker, F. Grass, H. Mignot, D. Hahnloser

**Affiliations:** 1 Department of General, Visceral, Thoracic, Transplantation and Pediatric Surgery, University Hospital Schleswig-Holstein, Campus Kiel, Kiel, Germany; 2 Department of Gynecological Surgery, Groupe Hospitalier Saintes Saint-Jean-d’-Angély, Saintes, France; 3 Department of Visceral Surgery, University Hospital Lausanne CHUV, Lausanne, Switzerland; 4 Kurt-Semm Center for Laparoscopic and Robotic Surgery, University Hospital Schleswig-Holstein, Campus Kiel, Kiel, Germany

**Keywords:** DEXTER^®^ Robotic Surgery System, Minimally invasive surgical procedures, Right colectomy, Robotic-assisted surgery

## Abstract

**Background:**

Robotic surgery has been increasingly used in colorectal surgery, where the improved visualization and dexterity are of benefit. The aim of this study was to confirm the perioperative and early postoperative safety and evaluate the clinical performance of the DEXTER^®^ Robotic Surgery System in right colectomy.

**Methods:**

A prospective, multicenter, post-market study was conducted across three sites in France, Germany, and Switzerland. Four surgeons performed the surgeries with DEXTER. The primary endpoints were major complications defined as Clavien–Dindo grades III–V, and procedural success rate. Secondary endpoints included perioperative safety and performance assessment up to 30 days post procedure.

**Results:**

A total of 33 patients with a median age of 71 years (IQR 63–78) and BMI 25.4 kg/m^2^ (IQR 23.4–27.9) were enrolled. Robotic intracorporeal anastomosis was performed in 49% of the procedures. All procedures were completed without device-related conversion to open surgery. Two patients experienced Clavien–Dindo III–V complications, none of which were device-related. Median estimated blood loss was 100 mL (IQR 20–120) with no blood transfusions. Operative time was 168 min (IQR 152–197) and length of stay was 5 days (IQR 4–6).

**Conclusions:**

Right colectomy with DEXTER is feasible and the results of this study support the short-term safety and clinical performance of the device, even early in the learning curve. Studies with long-term follow-up and functional outcomes are needed to assess long-term safety and performance.

**Clinical trial registration:**

ClinicalTrials.gov NCT05537727 (registered on 8 September 2022).

## Introduction

Since its introduction to colorectal surgery in 2002, robotic assistance has been increasingly adopted, especially for right-sided colon cancer [1]. Compared to conventional laparoscopy, robotic platforms offer enhanced three-dimensional visualization, tremor elimination, and increased instrument precision. Robotic surgery also offers improved surgeon ergonomics, potentially contributing to better outcomes [2]. These advantages of robotic systems translate into a reduced risk of complications compared to open and traditional laparoscopic procedures [3].

The benefits of robotic assistance are particularly noticeable during intracorporeal anastomosis steps, providing the necessary dexterity and control. While technically more demanding than extracorporeal techniques, intracorporeal anastomosis is associated with smaller incisions, lower conversion rates, and faster patient recovery [4].

Despite these benefits, the adoption of robotic surgery in colorectal practice remains limited by a number of critical implementation challenges. Traditional robotic platforms are associated with high acquisition and maintenance costs, the need for dedicated infrastructure and space in the operating room (OR) and adequate sterilization processes, limited availability across surgical units, complex training pathways [5], and workflow disruptions [6]. These barriers can limit widespread access and delay integration into existing surgical teams and protocols.

To overcome the aforementioned barriers, the DEXTER^®^ Robotic Surgery System was developed as an open-architecture robotic platform, compatible with existing OR infrastructure, including visualization systems and devices [7]. Its small, mobile design allows placement in any standard OR and effortless transfer between rooms, supporting access across multiple specialties. Unlike conventional systems, DEXTER is designed to seamlessly integrate into laparoscopic workflows, by preserving familiar trocar setups and allowing continued use of existing instruments such as laparoscopic staplers, making it easy to learn. It enables flexible intraoperative switching between laparoscopic and robotic techniques owing to its sterile surgeon console.

Since receiving the CE mark in 2020, DEXTER has been adopted across various surgical disciplines, including gynecology [8], urology [9], and general surgery [10–13]. Preliminary reports have confirmed its feasibility and benefits in colorectal procedures [14–16], supporting its potential as a solution that combines the advantages of robotics with the flexibility of laparoscopy.

The present study aimed to document the perioperative and early postoperative safety and clinical performance of DEXTER in robotic-assisted right colectomy within a post-market clinical follow-up (PMCF) framework following CE mark approval.

## Methods

### Study design

This was a prospective, single-arm, multicenter, multinational study conducted as part of a PMCF evaluation of DEXTER. The PMCF included three surgical indications, hysterectomy, partial nephrectomy, and right colectomy, with the present manuscript focusing on the robotic-assisted right colectomy cohort.

The study was carried out across three European hospitals: University Medical Center Schleswig–Holstein, Campus Kiel (Kiel, Germany), Groupe Hospitalier Saintes – Saint-Jean-d’Angély, (Saintes, France), and University Hospital Lausanne CHUV (Lausanne, Switzerland). The primary objective was to confirm the perioperative and early postoperative safety and the efficacy of using DEXTER in robotic-assisted right colectomy. Safety was assessed by the occurrence of postoperative complications classified as Clavien–Dindo grade ≥ III, recorded up to 30 days postoperatively. The efficacy was characterized by successful performance defined as the surgery being completed without any device-related permanent conversion to open surgery or fully laparoscopic surgical approach. A device-related conversion to open was defined as any incision larger than that required for specimen extraction due to limitations related to the device.

Adverse events were reviewed and adjudicated by an independent clinical event committee (CEC). Ethics committee approval was obtained in accordance with local requirements at each participating center. The study was registered on ClinicalTrials.gov (NCT05537727) and conducted in accordance with ISO 14155:2020 and the principles outlined in the Declaration of Helsinki.

All procedures were performed by four surgeons with prior experience in robotic-assisted surgery, three with at least 5 years of experience with other platforms and the fourth with approximately 75 previous procedures performed with DEXTER. Each surgeon completed the full DEXTER training program, comprising online didactic content, simulator training, and hands-on experience in dry and wet labs, before initiating clinical cases. At the time of study participation, most surgeons were still within their learning curve for right colectomy with DEXTER.

### Patient population

Eligible participants were adult patients (aged 18 years or older) diagnosed with suspected colon cancer and scheduled for robotic-assisted right colectomy using DEXTER. All patients were required to provide informed consent in accordance with local regulatory requirements and agreed to complete a 30-day postoperative follow-up period.

General exclusion criteria included morbid obesity (BMI ≥ 40), any absolute or relative contraindications to minimally invasive surgery (MIS), known bleeding diathesis, pregnancy, presence of a pacemaker or defibrillator, and the need for planned concomitant surgical procedures. Procedure-specific exclusion criteria included prior major abdominal or pelvic surgery and T4-stage colon cancer.

### The DEXTER® Robotic Surgery System

The DEXTER Robotic Surgery System (Distalmotion SA, Epalinges, Switzerland) consists of four modules: an open surgeon console, two patient carts with robotic instrument arms, and an endoscope cart with a robotic endoscope arm that can accommodate any endoscope used for laparoscopic procedures. The system offers five articulated single-use robotic instruments, including a monopolar hook, monopolar scissors, a bipolar Maryland dissector, a bipolar Johann grasper, and a needle driver. All instruments are fully articulated, offering seven degrees of freedom, and are compatible with 8-mm translucent trocars. The robotic system, including the surgeon console, is fully draped and maintained sterile throughout the procedure, allowing the surgeon to access the patient immediately without leaving the OR to scrub. As an open platform, DEXTER is compatible with standard laparoscopic towers, including existing imaging, electrosurgical, and endoscopic systems.

### Surgical technique

All procedures were performed using DEXTER. Patients were positioned in a supine posture with a slight Trendelenburg tilt of 5–10° and a left lateral tilt of 5–10° to optimize exposure of the right colon.

**Fig. 1:**
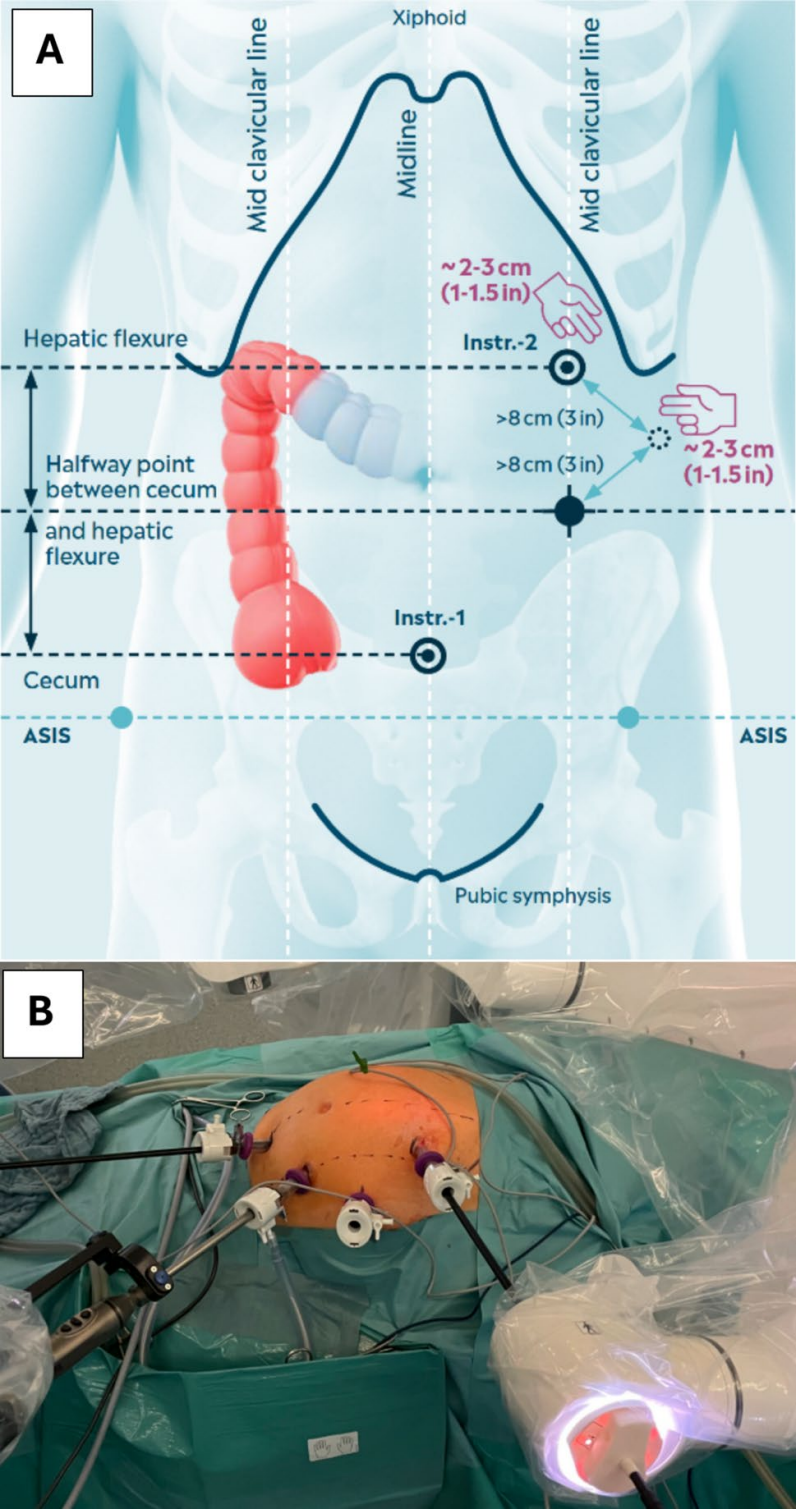
Trocar placement for right colectomy with DEXTER. **a** Manufacturer recommendations. **b** Trocar placement illustrated in one study case

Trocar placement generally followed manufacturer’s recommendations (Fig. 1): the endoscope port was positioned at the left midclavicular line, approximately midway between the cecum and hepatic flexure. The first instrument port was placed along the midline at the level of the cecum, while the second instrument port was centered at the midlevel of the hepatic flexure. An assistant port was inserted at a distance of at least 8 cm from both the endoscope port and the second instrument port. Trocar positioning was adapted as needed to accommodate individual patient anatomy.

The surgical procedure was divided into the following sequential steps: mobilization of fascial planes, lymphovascular dissection, bowel transection, anastomosis, specimen retrieval, and wound closure. Specific steps, such as stapling or suctioning, could be performed laparoscopically depending on surgeon preference or instrument availability, without workflow disruption. The surgical technique for ileocolic anastomosis was performed either extracorporeally or intracorporeally based on surgeon preference. Previously published studies provide information on the techniques used by some of the participating surgeons [14, 15]. For extracorporeal anastomosis, the mobilized bowel was extracted through a transumbilical incision, and either functional end-to-end or end-to-side anastomosis was performed. Intracorporeal anastomosis was performed using a Signia Stapling system (Medtronic plc, Dublin, Ireland) as previously described [15] or Echelon Flex™ Endopath™ (Johnson & Johnson, New Jersey, USA) or Endo GIA™ ultra universal stapler (Medtronic plc, Dublin, Ireland). An antimesenteric opening was created at both ends of the intestine, and an isoperistaltic side-to-side anastomosis was performed. The remaining opening was closed using a continuous double-row barbed suture (Stratafix™, Johnson & Johnson, New Jersey, USA). Specimen extraction was performed through a Pfannenstiel incision through the most caudal port.

### Data collection

Data were collected prospectively for all enrolled patients from the day of surgery through a 30-day postoperative follow-up period.

Operative time was defined as the duration from the initial skin incision to final skin closure (“skin-to-skin”). Console time referred to the cumulative duration during which DEXTER was actively in use. Docking time was defined as the time measured from the moment the patient carts began approaching the patient’s bed until the last incision pointer used for docking the instrument arms was removed, or the endoscope was fixed in the docked endoscope arm, whichever occurred later. A “switch” was defined as a change in modality between robotic and laparoscopic instrumentation during the procedure. As the surgeon console is sterile, the scrubbed surgeon could move between the operating table and surgeon console to perform both the robotic and laparoscopic steps without additional scrubbing in. Typically, the surgeries were assisted by a table-side assistant and a scrub nurse.

All adverse events were reviewed and adjudicated by an independent clinical event committee.

### Sample size and statistical analysis

A sample size of 30 was considered suitable to provide a reliable estimate of safety, as it allows calculation of a one-sided 95% confidence interval for observed complication rates, with upper bounds of 14.9% for a 3.3% rate (one event) and 23.9% for a 10% rate (three events), ensuring an acceptable level of precision.

Descriptive statistics were used in this study; median values with interquartile range (IQR) were used to present the data. Data were analyzed using StataCorp (2023. Stata Statistical Software: Release 18. College Station, TX: StataCorp LLC).

## Results

A total of 33 patients underwent robotic-assisted right colectomy using DEXTER between January 2023 and January 2025. The median patient age was 71 years (IQR 63–78), and the median body mass index (BMI) was 25.4 kg/m^2^ (IQR 23.4–27.9). The majority of patients (70%) were classified as American Society of Anesthesiologists (ASA) physical status I–II, while 30% were ASA III. Slightly more than half of the cohort (55%) were male. The majority of the patients presented an oncologic indication. Detailed demographic and baseline clinical characteristics are presented in Table 1.

**Table 1:** Patient characteristics

Parameter (*N* = 33)	Value
Age (years), median (IQR)	71 (63–78)
BMI (kg/m^2^), median (IQR)	25.4 (23.4–27.9)
Gender, male/female, *n* (%)	18/15 (55/45)
ASA score, *n* (%)	
I	4 (12)
II	19 (58)
III	10 (30)
Indications for surgery, *n* (%)	
Malignant	28 (85)
Benign^a^	5 (15)
Comorbidities, *n* (%)	
Hypertension	15 (39)
Diabetes	5 (15)
Dyslipidemia	5 (15)

^a^Preoperative suspicion of malignancy that was not confirmed after histological processing

Procedural success without device-related permanent conversion to laparoscopy or open surgery was achieved in all 33 cases (100%). While two procedures (6%) required conversion to open surgery, none were attributed to device malfunction or limitations. The two conversions were patient-related, one due to complex anatomy and one due to the discovery of a T4 tumor during the procedure, which is a contraindication for laparoscopic surgery. In four cases, the surgeons performed extracorporeal anastomosis via a mini-laparotomy, consistent with their standard practice. In the remaining patients, intracorporeal anastomosis was performed.

Operative outcomes showed a median skin-to-skin operative time of 168 min (IQR 152–197) and a median console time of 90 min (IQR 53–119). Estimated blood loss was low, with a median of 100 mL (IQR 20–120). Robotic assistance was employed across several key procedural steps: central lymphovascular dissection in 91% of cases, mesenteric transection in 65%, medialization of the colon and ileum in 52%, bowel transection in 58%, and anastomosis in 50%. For certain steps, particularly bowel or mesenteric transection and anastomosis, surgeons chose to temporarily switch to laparoscopic instrumentation such as staplers, putting DEXTER in its integrated laparoscopic mode. Such instances were performed based on surgeon preference or procedural efficiency. Overall, 58% of the cases were performed with zero or one modality switch between robotic and laparoscopic mode. The median time per switch was 35 s (IQR 20–45).

Three major complications classified as Clavien–Dindo grade ≥ III occurred in two patients (6%). The first patient suffered a trocar-site hernia in the right mid abdomen, which was discovered on postoperative day 4 and managed by laparoscopic surgical repair. The same patient developed an anastomosis leakage on postoperative day 9, and was reoperated with a segmental bowel resection followed by a side-to-side ileotransversotomy. The second patient suffered from a postoperative ileus and sepsis (Clavien–Dindo IIIa) that resolved within the study period.

The median length of hospital stay was 5 days (IQR 4–6). One patient (3%) required reoperation, and one (3%) was readmitted within 30 days following surgery (as mentioned above) (Table 2).

**Table 2:** Intra- and postoperative outcomes

Parameter (*N* = 33)	Value
Device-related conversions, *n* (%)	0
Patient-related conversions to open, *n* (%)	2 (6)
Planned mini-laparotomies for extracorporeal anastomosis, *n* (%)	4 (12)
Operative time (skin to skin) (min), median (IQR)	168 (152–197)
Docking time (min), median (IQR)	6 (4–8)
Console time (min), median (IQR)	90 (53–119)
Number of modality switches, *n* (%)	
0	2 (6)
1	17 (52)
2	1 (3)
3	13 (39)
Time per modality switch (s), median (IQR)	35 (20–45)
Robotic anastomosis, *n* (%)	16 (49)
Blood loss (mL), median (IQR)	100 (20–120)
Blood transfusions, *n* (%)	0
Length of hospital stay (days), median (IQR)	5 (4–6)
Number of serious complications, *n* (%)	
Clavien–Dindo grade IIIa	1 (3)
Clavien–Dindo grade IIIb	2 (6)
Clavien–Dindo grade IV–V	0
Readmission, *n* (%)	1 (3)
Reoperation, *n* (%)	1 (3)

## Discussion

This study represents the first prospective multicenter clinical investigation of the DEXTER^®^ Robotic Surgery System in robotic-assisted right colectomy. The procedures were performed by four surgeons, all of whom were in the early stages of their learning curve for performing right colectomy with the DEXTER system. Published data suggest that the learning curve for robotic right colectomy ranges from 15 to 68 cases [17–19]. Despite limited prior experience with DEXTER in right colectomy, all procedures in this cohort were completed successfully, without any device-related permanent conversions to laparoscopy or open surgery.

In our study, unplanned conversion to open surgery occurred in two (6%) cases, with both instances being patient-related. These findings compare favorably to existing literature on other robotic systems, where conversion rates to open surgery of up to 8.3% have been reported [20–22].

The three major complications (Clavien–Dindo grade ≥ III) observed in two patients were not specifically related to the use of DEXTER. The patient presenting with a trocar site hernia had several predisposing factors such as advanced age, relatively high BMI (27.8), and prior surgeries. The rate of major complications in our study falls within the range reported in the literature for robotic right colectomy, which varies from 6.2% to 14.3% [23–25], indicating a favorable safety profile even during the early learning phase.

Operative times (median 168 min) were comparable to those reported for other robotic systems, where operative durations ranged from 110 to 285 min [21, 26–28]. The estimated median blood loss was 100 mL, consistent with previously published ranges of 36.9–200 mL [26, 29, 30], and none of the patients required a blood transfusion.

With the median length of hospital stay of 5 days, our results fall within the reported range of 3–8.3 days for similar procedures [24, 26, 28, 30]. Only one patient (3%) was readmitted within 30 days, due to worsening metastatic disease.

The results of this study highlight several key strengths of DEXTER. The DEXTER system’s modular design allowed for intraoperative flexibility, enabling surgeons to alternate within seconds between robotic and laparoscopic approaches during the procedure without redocking, which contributed to procedural safety. Steps such as central lymphovascular dissection were predominantly performed robotically (91%), while other steps, including mesenteric transection, bowel transection, and anastomosis, were completed either robotically or laparoscopically depending on surgeon preference and efficiency. Laparoscopic staplers, energy devices (e.g., LigaSure™), and other standard laparoscopic instruments were used during the procedure without the need for workflow interruption, owing to the open-platform design of DEXTER and its full compatibility with existing OR equipment. This flexibility allowed surgeons to choose their approach according to personal preference, experience with the system, and case complexity. Additionally, the open console design supported barrier-free communication and effective team coordination, which have been positively associated with surgical performance [31]. These features underscore the system’s capacity to maintain safety while enhancing workflow integration.

Modality switching between robotic and laparoscopic instruments was both frequent and highly efficient, with a median transition time of only 35 s. The design of the DEXTER robot allowed the surgeon to remain scrubbed and within the operative field throughout the procedure, thereby enabling immediate access to the patient without requiring redocking or repositioning.

Regarding anastomosis, 49% of cases were completed robotically, reflecting the system’s instrumental precision and surgeon confidence, even during the early phases of the learning curve. The ability to switch rapidly between robotic and laparoscopic procedure proved especially advantageous during this initial adoption period, allowing surgeons to retain their preferred anastomosis techniques, while gradually increasing the proportion of robotic steps performed as the surgeon gains confidence with the system [15]. In comparison, literature shows varying adoption of intracorporeal anastomosis among surgeons using other robotic platforms, suggesting that system design and familiarity significantly influence technique choice [22, 32].

Nonetheless, this study has several limitations. The single-arm design, limited sample size, and short follow-up period restrict the generalizability of the findings. Additionally, key outcome measures such as histopathological margins and postoperative functional outcomes were not captured, which prevents the evaluation of early oncologic endpoints and full clinical performance. As a result of the small number of cases, which were also generated across three hospitals, the resection strategy is inconsistent, and the performance of complete mesocolic excision and D3 lymphadenectomy varies. Therefore, this study focuses on feasibility rather than oncopathological findings or oncological outcome measures.

Future research should focus on larger, comparative studies to validate these early findings and explore the broader clinical and economic implications of implementing DEXTER in colorectal surgery. A longer follow-up will be necessary to document the long-term safety, oncologic, and functional outcome measures.

## Conclusion

All procedures in this first prospective multicenter study were completed successfully, with no permanent conversions due to device-related issues, and a low rate of major complications. These findings indicate that DEXTER allows to perform a safe robotic-assisted right colectomy for suspected colon cancer. The system’s performance is consistent with outcomes reported for other established robotic platforms. Its primary advantage lies in its seamless integration into the existing laparoscopic OR workflow, facilitating a smoother transition to robotic surgery without disrupting established practices. While these initial findings are promising, larger prospective cohort studies are needed to provide more definitive evidence and assess long-term clinical outcomes.

## Data Availability

The data that support the findings of this study are not openly available due to reasons of sensitivity and are available from the corresponding author upon reasonable request.
